# β-Cell Pathophysiology: A Review of Advanced Optical Microscopy Applications

**DOI:** 10.3390/ijms222312820

**Published:** 2021-11-26

**Authors:** Gianmarco Ferri, Luca Pesce, Marta Tesi, Piero Marchetti, Francesco Cardarelli

**Affiliations:** 1 NEST Laboratory, Scuola Normale Superiore, Piazza San Silvestro 12, 56127 Pisa, Italy; gianmarco.ferri@sns.it (G.F.); luca.pesce1@sns.it (L.P.); 2 Islet Cell Laboratory, Department of Clinical and Experimental Medicine, University of Pisa, 56127 Pisa, Italy; marta.tesi91@gmail.com (M.T.); piero.marchetti@med.unipi.it (P.M.)

**Keywords:** β-cell, fluorescence, biophysics, insulin granule, diabetes, metabolism, NADH, tracking, FLIM

## Abstract

β-cells convert glucose (input) resulting in the controlled release of insulin (output), which in turn has the role to maintain glucose homeostasis. β-cell function is regulated by a complex interplay between the metabolic processing of the input, its transformation into second-messenger signals, and final mobilization of insulin-containing granules towards secretion of the output. Failure at any level in this process marks β-cell dysfunction in diabetes, thus making β-cells obvious potential targets for therapeutic purposes. Addressing quantitatively β-cell (dys)function at the molecular level in living samples requires probing simultaneously the spatial and temporal dimensions at the proper resolution. To this aim, an increasing amount of research efforts are exploiting the potentiality of biophysical techniques. In particular, using excitation light in the visible/infrared range, a number of optical-microscopy-based approaches have been tailored to the study of β-cell-(dys)function at the molecular level, either in label-free mode (i.e., exploiting intrinsic autofluorescence of cells) or by the use of organic/genetically-encoded fluorescent probes. Here, relevant examples from the literature are reviewed and discussed. Based on this, new potential lines of development in the field are drawn.

## 1. Introduction

β-cells localized in the pancreatic islets of Langerhans exert the unique function of producing, storing and secreting insulin, the peptide hormone that primarily regulates circulating glucose levels [1,2]. Their function is modulated by a complex interplay between nutrient, hormone and neuronal signals, which allows matching the release of insulin with the flexible needs of the body [2,3]. β-cell failure is the hallmark of diabetes [3,4,5], leading to hyperglycemia, which substantially contributes to the development of the acute (diabetic ketoacidosis, hyperosmolar hyperglycemic state) and chronic complications of diabetes (retinopathy, nephropathy, neuropathy, coronary heart disease, peripheral vascular disease), causing reduced life expectancy [6]. 

A peculiarity of the β-cell is the tight coupling between glucose stimulation (input) and insulin secretion (output) exerted by means of a cascade of highly regulated biochemical processes [3,7,8,9]. In brief, extracellular glucose molecules are first taken up by the β-cell through the GLUT-2 transporter. Once intracellular, glucose is readily phosphorylated and metabolically digested through glycolysis and the products of glucose oxidation, such as pyruvate and Nicotinamide Adenine Dinucleotide in reduced form (i.e., NADH), are shuttled together with other substrates into mitochondria by the mitochondrial glycerol phosphate dehydrogenase (mGPDH) and the mitochondrial malate dehydrogenase (mMDH) transporters [10] to participate to the Krebs’ cycle and produce large quantities of NADH [8]. NADH then acts as a potent electron carrier (being oxidized into NAD+) during mitochondrial oxidative phosphorylation, fueling the production of adenosine triphosphate (ATP) molecules. Increased ATP/ADP ratio levels induce closure of plasma-membrane-associated ATP-sensitive K^+^-channels, which, in turn, induces depolarization of the plasma membrane and activation of voltage-sensitive calcium channels. Ca^2+^ influx finally promotes and sustains insulin secretion, starting from the mobilization of the insulin secretory granules (ISGs) of the readily releasable pool (RRP) located near the plasma membrane, and then with the mobilization of ISGs located far from the plasma membrane using the microtubules as tracks. As mentioned above, this peculiar scheme of processes can be altered, at any level, both in type 1 and type2 Diabetes (T1D, T2D) with the final failure and/or loss of insulin-secreting β-cells [5,11].

Addressing such a complex realm of processes in living matter is a multifaceted challenge, mainly due to the lack of noninvasive and quantitative analytical tools. In particular, methods able to address molecular behavior at the proper temporal and spatial resolution in unperturbed (or minimally perturbed cells) are highly desirable. In fact, in most cases, information in one of the two dimensions (space or time) must be discarded. For instance, by using standard tools of quantitative chemistry (e.g., an ELISA assay) the amount of insulin released following glucose stimulation can be measured. This is an example of pure temporal information, with complete absence of spatial information. Conversely, by standard high-resolution microscopy (e.g., electron microscopy), very detailed spatial information can be retrieved, resolving even the structure of single insulin-containing vesicles. In this case, however, high spatial resolution is achieved at the expenses of temporal information: the cell is fixed. In between these two limit approaches, optical microscopy can represent a compromise. At the wavenumbers of visible or infrared light, in fact, cells can be interrogated, in principle, both in space and time. The question now is: how much of the β-cell “black box” can be illuminated?

**Figure 1:**
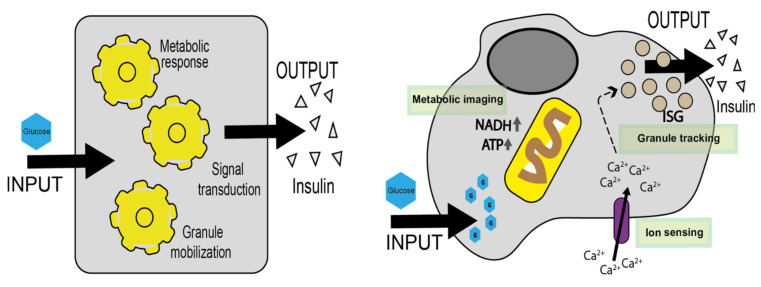
The β-cell as a black box. Main functions (metabolic response, signal transduction and granule mobilization) of the β-cell that link glucose stimulation (input) to insulin secretion (output) are reported as ‘black boxes’ in the image on the left. In detail, on the right, are the most important molecular players that exert these functions. Labelled in green are three microscopy optical tools compatible with living matter and capable of monitoring these functions in real time. ISG, insulin secretory granule.

To simplify, let us divide the complex machinery of events connecting the input to the output in β-cells into three major sections: (i) glucose uptake and processing through metabolism, (ii) second-messenger production, including ion fluxes, and (iii) mobilization/exocytosis of ISGs (Figure 1). The arsenal of biophysical techniques available at present holds the potential to tackle these tasks directly in living matter. In this review, we collect the most relevant applications of optical microscopy-based biophysical tools and discuss new potential lines of development in the field.

## 2. Optical Microscopy to Study Glucose Metabolism in Β-Cells

For the aim of studying glucose metabolism in living cells, techniques capable of not altering the chemical identity and endogenous stoichiometry of the key molecular players are fundamental. Standard molecular-based biochemical assays (e.g., Western blot and gene expression), although very useful, imply studying molecular processes outside of the natural context of the living cell.

To tackle this issue, the use of endogenous molecules, naturally present in the cellular context, which allow a readout of metabolic activity, is possible. Among them, NAD(P)H species have historically been used as intrinsic metabolic biomarkers in a context of label-free approaches. In fact, given its biological importance, especially in the β-cell metabolic response to glucose, the possibility to monitor intrinsic NAD(P)H level in living cells could represent a valid marker for qualitative/quantitative assessments on cellular metabolic state. In this sense, the pioneering studies of Chance et al. [12,13] have laid the ground for future works based on NAD(P)H optical properties. In fact, both oxidized NAD(P)+ and reduced NAD(P)H show strong absorption in the UV region around 260 nm; however, only NAD(P)H absorbs appreciably at 350–365 nm and emits fluorescence with a peak at 460 nm. In the 400–500-nm emission interval the contribution of other autofluorescent species is negligible, except for collagen, which is not present in laboratory-cultured cells [14]. Among intracellular intrinsic fluorophores, NAD(P)H also shows good two-photon cross-section if excited at around 720 nm [15], allowing two-photon excitation and, therefore, achievement of a good penetration depth and minimal perturbation/damage of the sample.

**Figure 2:**
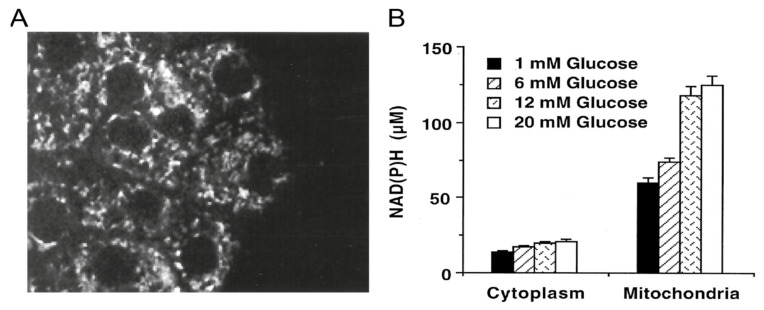
NAD(P)H intensity analysis. (**A**) Two photon microscopy of NAD(P)H autofluorescence of a mouse islet. (**B**) Quantification (in μM) of NAD(P)H abundance in cytoplasmic and mitochondrial regions for different glucose concentrations. Adapted from Patterson et al. [16] (Copyright (2000) National Academy of Sciences, USA).

Two photon NAD(P)H fluorescence imaging was applied to the specific context of the β-cell by Patterson et al. [16] to characterize the response in terms of NAD(P)H fluorescence-intensity signal to glucose-stimulation in rodent Langerhans islets, with cellular and subcellular resolution (Figure 2).

In Haythorne et al. [17], NAD(P)H intensity was used as an indicator for mitochondrial activity in control and T2D mice islets, revealing an attenuated metabolic response in terms of NAD(P)H production to glucose stimulation in the latter. Yet, although undoubtedly informative, intensity-based measurements may contain artifacts due to the heterogeneity of intracellular NAD(P)H concentration and to the differing quantum yields (QY) of the NAD(P)H molecules which are bound to enzymes (typically high QY) as compared to those which are free in the cytoplasm/nucleus (typically with low QY). Interestingly, however, the NADH free and bound forms have different fluorescence lifetimes. The free form is mainly in folded conformation and, due to self-quenching, has a fluorescence lifetime about 10-fold shorter than the extended conformation that is characteristic of NAD(P)H bound to enzymes. Based on this, it appears clear that the fluorescence lifetime of NAD(P)H can be used as a concentration-independent, label-free tool to monitor the ratio of free and bound NAD(P)H in living cells. Fluorescence Lifetime IMaging (FLIM) microscopy, in particular, in which the “contrast” depends on the fluorescence lifetime and not on the local concentration and/or intensity of the fluorophore, was introduced more than 30 years ago by Lakowicz and collaborators [18,19]. As a further step, FLIM was combined with a graphical, fit-free procedure of data analysis known as phasor analysis [20]. A distinguishing feature of phasor analysis is that any region of the phasor plot (which corresponds to a type of lifetime decay) can be selected with cursors to directly identify the pixels in the image with that given lifetime. This process can be also reversed, and different areas of the image can be selected to highlight the corresponding distribution of points in the phasor plot. As such, the phasor approach to FLIM provides a powerful tool for visualization of data and for fit-free analysis of the information contained in the thousands of pixels constituting the image. Graphically, in the phasor plot it is possible to define a ‘metabolic trajectory’, which starts from a region of the phasor plot corresponding to the lifetime characteristics of NAD(P)H bound to enzymes (e.g., from 3 to 3.6 ns) [21,22] and ends in a region corresponding to the lifetime of NAD(P)H free in solution (e.g., around 0.4 ns). Once the ‘metabolic trajectory’ is defined, data can be treated according to their position with respect to the trajectory; for instance, by applying a specific lookup table (LUT). The output of the analysis is then a color-coded image in which each pixel contains information on the ratio between bound and free NAD(P)H molecules [23]. In turn, the ratio of bound/free NAD(P)H species can be used to identify different metabolic states of cells (e.g., the glycolytic phenotype, with a low bound/free ratio, and the oxidizing condition, with a high bound/free ratio) [24].

**Figure 3:**
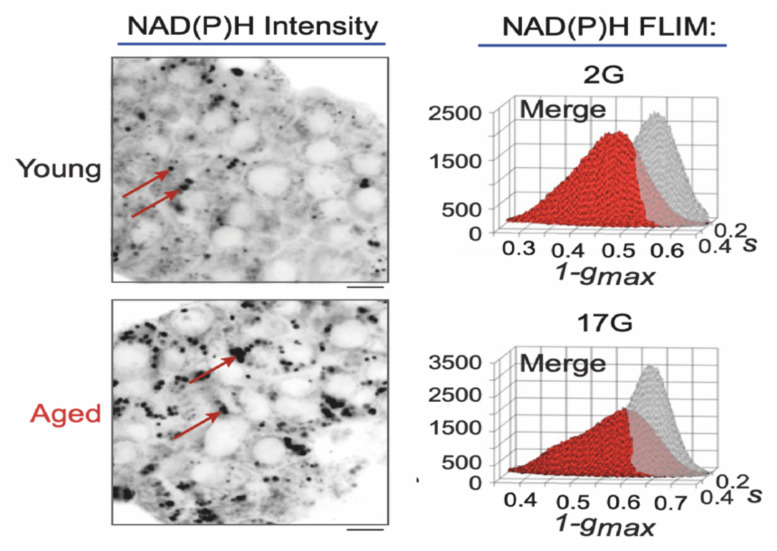
Phasor-FLIM analysis on human islets. Multiphoton FLIM of NAD(P)H in human islets from representative young (24 years old donor) and aged (58 years old donor) human donors. Phasor histograms show the frequency distribution of NAD(P)H lifetimes in young (black) and aged (red) islets. Scale bar = 5 μm. 2G, 2mM glucose. 17G, 17mM glucose. Adapted from Gregg et al. [25].

Specifically in the context of insulin secretion, phasor-FLIM analysis on NAD(P)H species was used to monitor the metabolic status of intact human-derived and mouse-derived Langerhans islets under different stimuli [25,26,27]. In particular, in the work by Gregg and collaborators [25], phasor-FLIM analysis revealed an increase in the ratio of bound/free NAD(P)H species in human and mouse islets in response to glucose stimulation; an effect then impaired by aging (Figure 3). The observed shift in NAD(P)H lifetimes measured on entire islets after glucose stimulation is generally attributed to the response of the β-cells to this event, although in the literature available so far, the different endocrine cell types within the islet were not identified.

**Figure 4:**
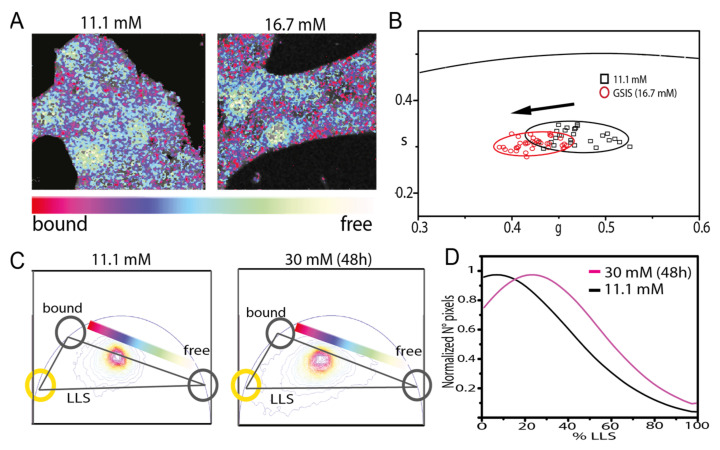
Phasor-FLIM analysis on INS-1E cells. (**A**) Example images of NAD(P)H lifetime (bound and free) in INS-1E cells in 11.1mM and 16.7 mM glucose, colored in accordance with the color bar defined below. (**B**) Scatter plot of the average values of distinct phasor distributions, each relative to distinct acquired cells. Black squares represent cells in maintenance condition, and red circles stimulated cells. (**C**) Three components analysis on phasor plot of cells cultured in 11.1 mM glucose and exposed to high glucose medium for 48 h. (**D**) Resultant graph from three components analysis, in which percentage of LLS species is plotted vs the number of pixels and long lifetime species are present for the aforementioned conditions. LLS, long lifetime species. GSIS, glucose stimulated insulin secretion (16.7 mM glucose). Adapted from Ferri et al. [28].

Recently, some of us [28] investigated the specific metabolic behavior of β-cells upon glucose stimulation using a rat-derived model of β-like cells, the Insulinoma 1E (INS-1E) cells, and a phasor-FLIM approach to NAD(P)H imaging (Figure 4). Analogous to data from an intact islet, in this model cells showed a metabolic shift towards oxidative phosphorylation (in terms of bound/free NAD(P)H). 

**Figure 5:**
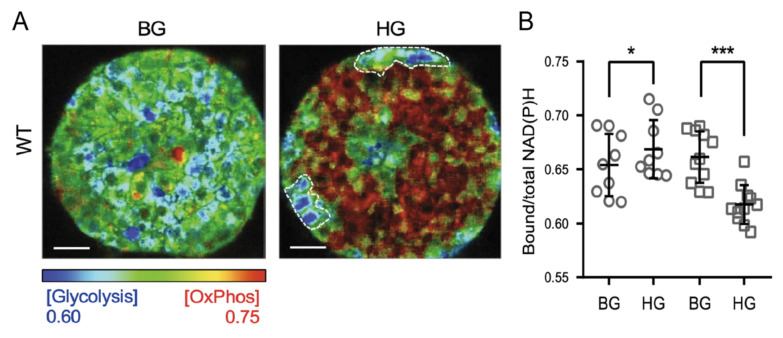
Phasor-FLIM analysis on murine islets. (**A**) FLIM images depicting relative glycolysis and oxidative phosphorylation in islets from a wild-type mouse islet at basal and high glucose. (**B**) Panels show bound/total NAD(P)H value for β cells and α cells, respectively, in WT mouse islets at BG and HG. Scale bar: 10 µm. * *p* <  0.05, *** *p* <  0.001. WT, wild type; BG, basal glucose (4 mM); HG, high glucose (16 mM). Adapted from Wang et al. [29].

Interestingly, the metabolic shift towards oxidative phosphorylation after glucose stimulation observed in β-like cells was recently confirmed in the work by Wang and collaborators [29]. Here, the single-cell resolution typical of the phasor-FLIM approach was exploited to distinguish the metabolic signature of β- and α-cells in intact murine islets (Figure 5) [29]. The authors, based on obtained results, suggest that in healthy cells glucose enhances oxidative phosphorylation in β-cells and suppresses oxidative phosphorylation in α-cells. In Type 2 diabetes, instead, glucose increases glycolysis in β-cells, and only partially suppresses oxidative phosphorylation in α-cells.

From a methodological point of view, phasor-FLIM has the potential to couple high spatial resolution (subcellular/cellular) with high temporal resolution (seconds/minutes), thus paving the way to the study of the complex spatiotemporal pattern of activation of cells within the islet in response to selected stimuli [30,31]. Being label-free, FLIM on NAD(P)H does not yield artifacts due to the alteration of the molecular stoichiometry but still suffers from a few specific limitations, namely: (i) NADPH and NADH species cannot be distinguished; (ii) the metabolic response is reported in terms of bound/free ratio but changes can occur in both the numerator and denominator of the measured ratio; (iii) the approach does not have native single-enzyme or single-pathway resolution.

## 3. Optical Microscopy to Study Second Messenger Production in β-Cells

**Figure 6:**
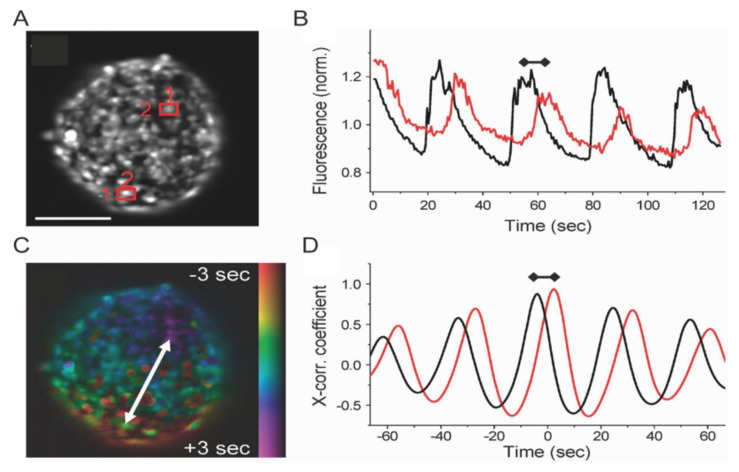
Ca^2+^ imaging with fluorescent biosensor. (**A**) Intensity image of Fluo-4-stained islet. Scale bar: 100 μm. (**B**) Time course of Fluo-4 intensity oscillations in the two cells highlighted in (A) (red rectangle). Oscillations of cell 1 (black line) precede those of cell 2 (red line). (**C**) False color scale phase map representing the propagation of the calcium wave across the islet from regions colored in red (early [Ca^2+^] increase) to regions colored in blue/purple (later [Ca^2+^] increase). (**D**) Cross correlation trace of cell 1 and 2. Difference in time between peaks represents the propagation of Ca^2+^ waves. Adapted from Benninger et al. [39].

The coupling between glucose-mediated metabolism activation and insulin secretion is mediated by a cascade of intracellular processes that starts with an increase in the ATP/ADP ratio, which causes the closure of ATP-sensitive K^+^ (K_ATP_) channels at the plasma membrane. The interruption of the outflow of K^+^ ions from the cell cytoplasm induces depolarization of the plasma membrane and promotes the influx of Ca^2+^ ions through voltage-dependent Ca^2+^ channels (VDCCs). Finally, Ca^2+^ acts as a second messenger and activates the exocytotic machinery, hence promoting the fusion of insulin-containing granules with the plasma membrane and insulin release into blood vessels [32]. In this complex scenario, biophysics has focused mainly on the study of Ca^2+^ fluxes and their spatiotemporal regulation in the β-cell in physiology and disease [33,34]. To this aim, organic fluorophores show interesting properties as Ca^2+^ sensors, being compatible with living matter and having the major advantage that they can be used either with laboratory cell lines, primary cells and intact islets without genetic manipulation. Among the different classes of fluorescent Ca^2+^ sensors, Ca^2+^ chelators are mostly used, with a number of different variants available and with different Ca^2+^ binding affinities and spectral properties [35]. Their compatibility with the intact Langerhans islet allowed probing the dynamics of Ca^2+^ waves upon glucose stimulation, revealing a difference in Ca^2+^-oscillation frequency between the first and second phase of insulin secretion [36,37]. In mice islets, coordination among β-cells through gap junctions has been investigated by the organic Ca^2+^-sensor Fluo-4 AM. The biophysical model originating from the data has highlighted the features of intra-islet Ca^2+^ oscillations, characterized by a variation of timings and starting points (Figure 6) [38].

Differences between Ca^2+^ modulation in mice and human islets have also been observed, with the first characterized by whole-islet waves [40] and the latter by local β-cell-cluster waves [41,42]. Although widely used, it is worth mentioning that inhibition of Na^+^/K^+^-ATPase by Ca^2+^ chelation has been reported [43], and since Na^+^/K^+^-ATPase contributes directly to a hyperpolarization of the β-cell membrane potential, this kind of biosensor could artificially increase cell electrical activity. Worthy of mention is that Broichhagen and co-workers recently attained optical control of insulin release using a photo-switchable sulfonylurea which targets the ATP-sensitive K^+^ (K_ATP_) channels and activates downstream Ca^2+^ release and exocytosis of insulin [44]. This study opens new potential avenues of development at the crossroad between biophysics, pharmacology, and medicine.

To conclude, genetically encoded fluorescent biosensors for ions, voltage changes or metabolic activity are available (e.g., fluorescent NADH sensors [45] and fluorescent ATP or cAMP sensors [46]). Still, their implementation is subordinated to the use of virus-based transduction technologies (to penetrate the islet) and hampered by the need to over-express the desired sensor, thus potentially altering the stoichiometry of the molecules of interest in the cell.

## 4. Optical Microscopy to Study Insulin-Secreting Granules

The β-cell metabolic response to glucose rapidly triggers insulin secretion by granule mobilization first and then granule fusion to the plasma membrane. An increasing amount of evidence show that a tight regulation of both ISGs structural (e.g., size) and dynamic (e.g., speed, diffusion mode) properties is pivotal to assure ISGs function, i.e., the proper regulation of glucose homeostasis through insulin secretion at the cellular, and then the systemic level [47]. In fact, defects in granule structural and dynamic properties are found as hallmarks of β-cell dysfunction and, in turn, of the onset of the pathological condition. For instance, it was recently proposed that hypercholesterolemia is capable of increasing granule size and, at the same time, impairing membrane trafficking properties [48]. As mentioned earlier, two limit approaches are able to yield quantitative information on granule structural and dynamic properties, although not simultaneously. On one side, the insulin-secretion process can be addressed by measuring the amount of insulin released following glucose stimulation by means of absorbed antibodies and colorimetric reactions (e.g., by an ELISA assay) [49]. Here, however, only indirect evidence about granule behavior can be obtained. For instance, based on ELISA assays, it was postulated that granule-dependent insulin release follows a biphasic pattern, with a first peak of secretion occurring after the glucose pulse due to a pool of ready-to-release granules, and a second broader peak of secretion at longer times, due to a pool of reservoir granules.

On the other hand, the high spatial resolution available by means of, for instance, Transmission Electron Microscopy (TEM), can be pivotal to address the finest structural properties of ISGs, but at the expense of any information about their dynamics [50,51,52]. A warning was recently published concerning TEM-derived granule structural information, as it can be prone to fixation artifacts [53]. In fact, chemical fixation with aldehydes induces morphological alterations due to dehydration of the sample which can, eventually, lead to errors in granule size estimation.

**Figure 7:**
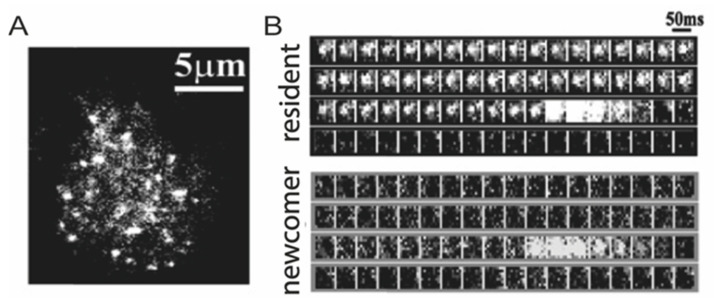
Exocytosis of ISGs studied by TIRF microscopy. (**A**) Example of TIRF image of GFP-labeled insulin granules in a rat primary β-cell. Scale bar: 5 μm). (**B**) Sequential images (1 μm × 1 μm per 50-ms interval) of a granule docking and fusing with the plasma membrane were presented during 22 mM glucose stimulation. Adapted from Ohara-Imaizumi et al. [54].

Between the two mentioned extremes, measurements performed by fluorescently labelling granules and studying their behavior in living cells, offer a valuable platform to increase our knowledge. Traditionally, optical-microscopy applications to ISGs properties entailed the use of either total internal reflection fluorescence (TIRF) imaging [54,55,56] or single particle tracking (SPT) studies [57,58,59,60,61] with a temporal resolution spanning between approximately 1 s [57] and 50 ms [58], depending on the specific application. TIRF is technically limited to study the cell/glass interface in adherent cells (Figure 7). In fact, it was selectively used to clarify the process of granule docking and fusion at the plasma membrane, and the impaired insulin release process during the first phase in diabetic Goto–Kakizaki rat β-cell [62]. Heaslip and coworkers visualized the dynamic and complex movement by TIRF of individual insulin granules before and after glucose stimulation, and subsequently pharmacological cytoskeletal treatments [58]. This was made possible by the use of fluorescently-labelled ISGs, achieved mainly through genetically-encoded fluorescent proteins (FPs) or fluorescent sensors capable of registering ions released into the extracellular medium (e.g., Zn^2+^ ions cosecreted with insulin [63]).

**Figure 8:**
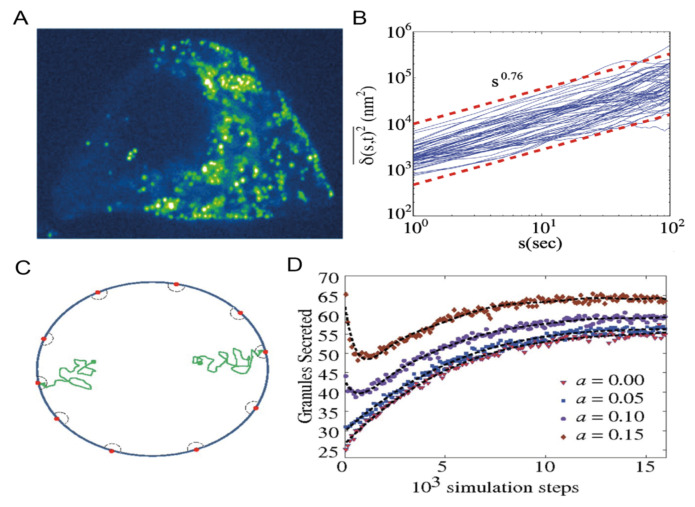
Characterization of ISGs mode of motion. (**A**) Typical confocal fluorescence image of syncolin-EGFP–labeled insulin granules in live MIN6 cells. (**B**) Time-averaged mean square displacement (TA-MSD) as a function of lag time. Blue lines are for individual trajectories of length t = 200 s, and the red dashed lines show an exponent of α = 0.76. (**C**) Simulation system scheme used to test biphasic secretion can be compatible with subordinated random walk. (**D**) Secretion flux of simulated granules. Adapted from Tabei et al. [57].

Tracking of ISGs, on the other hand, was performed by exploiting a number of intragranular chimeric FPs. For instance, neuropeptide Y (NPY)-FP and phogrin-FP were used in pivotal studies aimed at quantifying granule mean square displacement (MSD) under different stimuli [60,61,64]. Undoubtedly, SPT analysis was fundamental in extending the spatial scale of investigation of granule dynamics, allowing ISGs trafficking to be fully characterized in terms of diffusion modes in the cell cytoplasm. As a remarkable example, S.M.A. Tabei and coworkers [57] in 2013 analyzed SPT data on insulin granules expressing syncollin-EGFP in MIN6 cells (Figure 8A) to better understand the motions contributing to intracellular transport. They observed anomalous diffusion of labeled granules. Interpretation of such data is typically done either assuming particles working against fluctuating obstacles (fractional Brownian motion) or assuming the presence of a broad distribution of dwell times for traps (continuous-time random walk). However, the authors proved that statistical tests based on these two models give conflicting results. They proposed a different solution by introducing a subordinated scheme in which particles in cages with random dwell times undergo correlated motions owing to interactions with a fluctuating environment. (Figure 8B). They also related this picture to the underlying microtubule structure and provided a physical picture for how diverse pools of insulin granules can contribute to biphasic secretion (Figure 8C,D).

**Figure 9:**
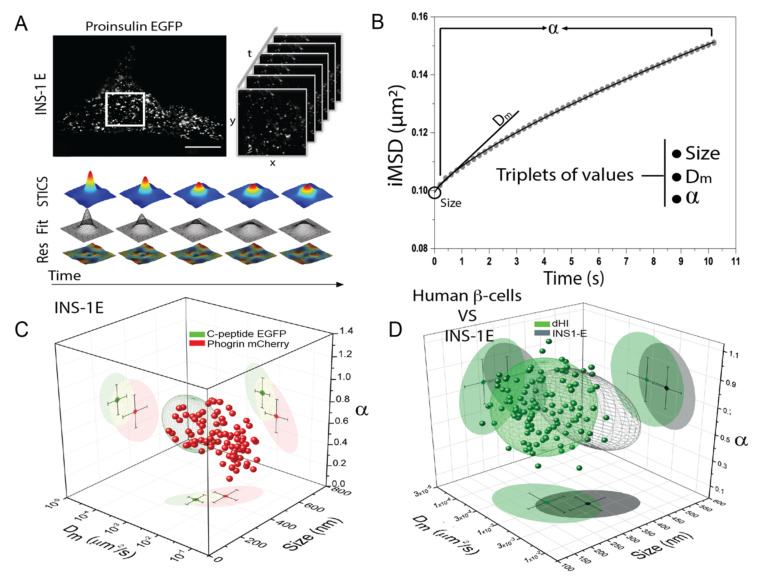
iMSD analysis on labelled ISGs in INS-1E and isolated human β-cells. (**A**) Example of INS-1E cell transfected with proinsulin-EGFP: the visible punctuated pattern represents the ISGs containing the GFP-tagged c-peptide fragment. iMSD analysis consists in a time lapse acquisition of a cytoplasmic region of the transfected cell. Spatio-temporal image correlation spectroscopy (STICS) analysis is then performed on the stack of images. (**B**) iMSD curve obtained by Gaussian fitting of the spatiotemporal correlation functions representing the average diffusion law of the whole population of captured ISGs. For each experimental curve, values of anomalous coefficient α, local diffusivity D_m_ and size are extracted by fitting procedures. (**C**) Structural and dynamic properties of ISGs labelled with proinsulin-EGFP (green) and phogrin-EGFP (red) represented as a scatter plot in which the three values of α, D_m_ and size of each acquisition are organized in a 3-D parametric space. (**D**) Comparison between syncollin-EGFP labelled granules in INS-1E cells (grey) and in dispersed human islet β cells (dHI). Adapted from Ferri et al. [71,73].

Interestingly, with specific fluorescent probes, ISGs have also been categorized in terms of mobility and spatial arrangement based on their age and maturation stage [65,66]. In this context, a fluorescence-based spatio-temporal fluctuation analysis named iMSD (*image derived* mean square displacement) was recently proposed by some of us as a powerful quantitative tool to simultaneously extract average structural and dynamic properties from labelled diffusing objects, either molecules [67,68] or entire subcellular nanostructures/organelles [69,70], directly from standard imaging. Contrary to SPT, the iMSD approach does not need complex instrumentation, preliminary assumptions on the system and extraction of single-object trajectories, thus being inherently fast and technologically easy to implement. By fitting the iMDS curve, a triplet of values describing the diffusion law of the object of interest can be retrieved. In particular, (i) the average size of diffusing objects, (ii) the local diffusivity Dmicro (D_m_) and (iii) the anomalous coefficient ‘α’ (α < 1 subdiffusive, α = 1 brownian and with α > 1 a superdiffusive behavior). In our approach, these values can be used to identify a unique point in a 3D parametric space, so that the clustering of single-cell data points depicts the overall structural and dynamic properties of the structure of interest [71]. The iMSD approach was recently applied by some of us to perform screening of ISGs labelled with different genetically-encoded fluorescent proteins both in INS-1E cells [71,72] and in cells disaggregated from human-derived islets [73] (Figure 9A,B). Concerning the application to INS-1E cells, a reference using cells expressing proinsulin fused to a FP (c-peptide EGFP) under basal culture conditions was created by iMSD analysis and then validated by testing well-established stimuli, such as glucose intake, cytoskeleton disruption, or cholesterol overload [71]. At this point, the effects of a plethora of FP-tagged ISG protein markers on the structural and dynamic properties of the granule was evaluated (i.e., synconllin-EGFP, IAPP-Emerald, phogrin-EGFP). While iMSD analysis produced similar results for most of the lumenal markers, the transmembrane marker phogrin-FP induced a substantial granule enlargement and higher mobility, together with a partial depolymerization of the actin cytoskeleton, and reduced cell responsiveness to glucose stimulation. These data suggest a more careful interpretation of many previous ISG-based reports in living β-cells [60,61,74,75] (Figure 9C). Concerning the application to disaggregated cells, iMSD was pivotal to define, for the first time, the structural/dynamic fingerprint of ISGs in human cells compared to immortalized rat-derived cells (Figure 9D). Then, iMSD analysis allowed probing of fingerprint variations under selected conditions of interest. It was shown that palmitate (a well-known lipotoxic agent) affects ISGs dynamics in response to acute glucose stimulation by abolishing the ISGs mobilization typically imparted by glucose and, concomitantly, by reducing the extent of ISGs active/directed intracellular movement. Finally, iMSD analysis highlighted quantitatively a protective effect against Palmitate induced by cotreatment with Exendin-4 (an agonist of the Glucagon-like peptide-1 receptor, GLP-1R, being used for the treatment of T2D). This latter is able to normalize ISG dynamics, i.e., re-establish ISG mobilization and ability to perform active transport in response to glucose stimulation. These data support the idea that GLP-1R agonism may exert its beneficial effect on human β-cells under metabolic stress by maintaining ISGs’ proper intracellular dynamics [73].

## 5. Future Directions

In spite of the crucial role played by β-cells in systemic glucose homeostasis, a detailed comprehension of the mechanisms regulating their metabolic response to blood glucose in physiology and disease is far from being reached. The task appears even more challenging if we consider the natural context in which the β-cell operates, i.e., the intact Langerhans islet. In this submillimetric organ, in fact, intracellular complexity and heterogeneity are accompanied by the intercellular aspects, since a vast range of both homotypic and heterotypic cellular connections are involved in the fine regulation of insulin secretion [76]. In addition, the presence of hundreds of cells tightly packed in a three-dimensional architecture makes it even more complicated to achieve single-cell identification and analysis, mainly because of poor light penetration. Even by using infrared wavelengths combined with multiphoton microscopy, the effective penetration-depth that can be achieved is not sufficient to perform imaging of the entire islet. This is due mainly to the refractive-index mismatch caused by the variety of biomolecules with different optical properties contained in the islet (e.g., lipids, fibers, and proteins), and to light scattering and/or absorption by the same molecules. A possible strategy to improve light penetration is represented by the use of tissue-clearing procedures [77,78,79].

**Figure 10:**
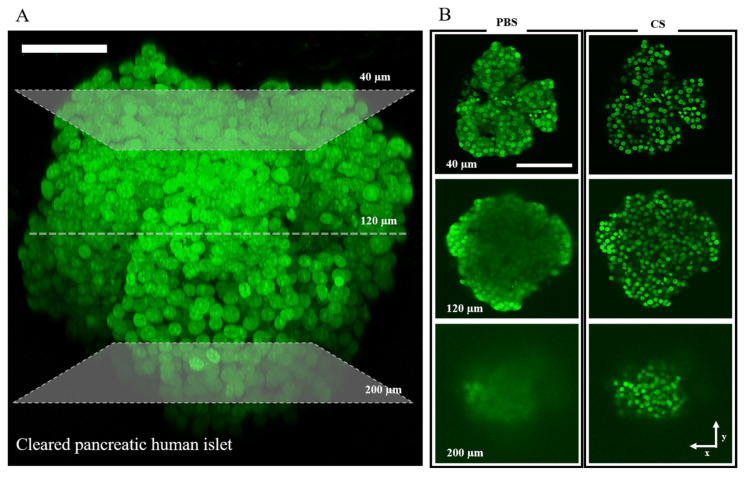
Clearing procedure applied on intact Langerhans islet. (**A**) 3D reconstruction of pancreatic islet labeled with DAPI using two-photon excitation microscopy combined with the clearing procedure (Exc. 800 nm; scale bar 50 µm). (**B**) XY images at different depths (40, 120, and 200 µm) in normal (PBS) and cleared sample (CS). After fixation with paraformaldehyde, the specimens were labeled with DAPI and then incubated with a solution consisting of a mixture of 2,2′-Thiodiethanol (TDE) and PBS. The ability of TDE to decrease the refractive index by dehydration allows reduction of the light scattering process, and performing whole-human islet imaging of cells (Exc. 800 nm; scale bar 100 µm).

Preliminary results obtained by us are encouraging along this line of experimental development (Figure 10). Of course, these procedures are not compatible with live-tissue analysis, as they become effective exclusively upon tissue fixation. Still, along with an increase in imaging performance, they offer the possibility of identifying the different cell types in the islet (e.g., by the use of antibodies) and can be combined with metabolic imaging (e.g., NADH lifetime analysis, as discussed above), this latter being, in principle, achievable either before or after tissue fixation/clearing. Such a multiplexed biophysical analysis, in which multiphoton microscopy on a cleared sample is combined with the use of either intrinsic or exogenous fluorescent probes, could represent a turning point for islet functional imaging in the near future. Besides light penetration, an increase in the nominal spatial resolution of imaging would also be desirable, given the high density of cellular structures densely packed in a few tens of micrometers of tissue. To this aim, the recent advent of super-resolution microscopy (SRM) opens intriguing new possibilities. Successful applications of SRM to isolated β-cells can be found in the literature.

For instance, Heaslip and coworkers determined how the spatial organization of actin and the tubulin cytoskeleton contribute to insulin trafficking in normal and pharmacologically perturbed state using a combination of stochastic optical reconstruction microscopy (STORM) and live cell TIRF microscopy [58]. Bogan and collaborators used structured illumination microscopy (SIM) to study the impact of cholesterol overloading on ISGs fine structure [48]. However, the performance of SRM is inevitably challenged by the use of a 3D sample, such as the intact islet, for the reasons discussed above. Intriguing future possibilities for super resolution in the islet, although technically demanding, may derive from the use of infrared light to perform stimulated emission depletion (STED)-based imaging, the feasibility of which was demonstrated (not in the islet) by Bianchini and co-workers [80]. An additional line of development could entail the use of expansion microscopy (ExM), a technique proposed in 2015 by E. Boyden and collaborators, which allows investigators to identify small structures by expanding them using a polymer system [81]. The premise is to introduce a polymer network into cellular or tissue samples, and then physically expand that polymer network using chemical reactions to increase the size of the biological structures [82]. Among other benefits, ExM allows those small structures to be imaged using standard equipment and a wide range of microscopy techniques [83]. Additionally, an exciting possibility using such methodologies is to improve the accessibility and the density of external probes (i.e., classic antibodies) in 3D structures such as human islets, exploiting a decrowding protein effect by the expansion process [84,85].

When research objectives must be pursued in a living tissue, however, alternative strategies must be considered for both fluorescent labelling and imaging. For instance, if the target of research is granule dynamics and regulation, a granule-specific fluorescent probe should be first delivered into the intact islet. To this end, lentiviral transduction systems already represent a valuable option, with a few examples in the literature [86,87]. These systems allow packaging, delivery and expression of genetically-encoded fluorescent proteins tagged to the markers of interest but remain inherently challenging in terms of safety issues and manipulation complexity. To overcome these latter issues, it is worth mentioning that a new-generation of membrane-permeable organic dyes with interesting applications in the context of islet imaging have been introduced recently [88,89]. For instance, a new Zn^2+^ chelator molecule, named Zigir, was proposed to label ISGs in dispersed cells and intact islets. However, in spite of its specificity for Zn^2+^ ions (which are abundant in the granule) Zigir also accumulates in other acidic organelles, such as lysosomes [88].

Based on these general concerns, a label-free option for granule imaging is being actively researched. Worthy of mention is that in a recent study conducted analyzing the light scattered by entire Langerhans islets, the ISG was proposed to be a reasonable candidate as intracellular source of scattered signals, possibly because of the densely-packed insulin semi-crystal that is present in the organelle lumen [90]. However, a detailed analysis of the light-scattering properties of single ISGs in isolated cells has partially refuted this hypothesis, demonstrating that other intracellular organelles in addition to ISGs are able to scatter light [91]. At present, these results prevent the use of scattering as a tool for specific granule imaging. An intriguing alternative possibility could be that of exploiting the intrinsic autofluorescence of selected aminoacidic residues, i.e., tyrosine, tryptophan and phenylalanine, to perform label-free granule imaging. In fact, the primary sequence of human insulin, for instance, comprises four tyrosine and three phenylalanine residues [92], while the primary sequence of glucagon comprises one tryptophan, two tyrosine and two phenylalanine residues. These residues can be excited by exploiting multiphoton microscopy and being able to collect the signal in the 280-380-nm range [93]. As an example, in the pioneering work by Maiti and coworkers, granules loaded with serotonin (chemically, an analogue of tryptophan) were imaged by means of three-photon microscopy in live neuronal cells [94]. The optical properties of these amino acid residues can be affected by several environmental factors such as temperature, pH, ionic strength, noncovalent interactions and, in general, are characterized by a low quantum yield. Still, it is worth noting that each ISG hosts approximately 2 × 10^5^ insulin molecules densely packed in hexamers, with an estimated final insulin concentration of about 40 mM within each granule^8^. The undoubted technological advances we are facing nowadays, especially in terms of detector sensitivity, could pave the way to new exciting possibilities for single-granule imaging, tracking, and analysis in live cells and tissues, with no need to over-express fluorescently-tagged granule proteins.

**Figure 11:**
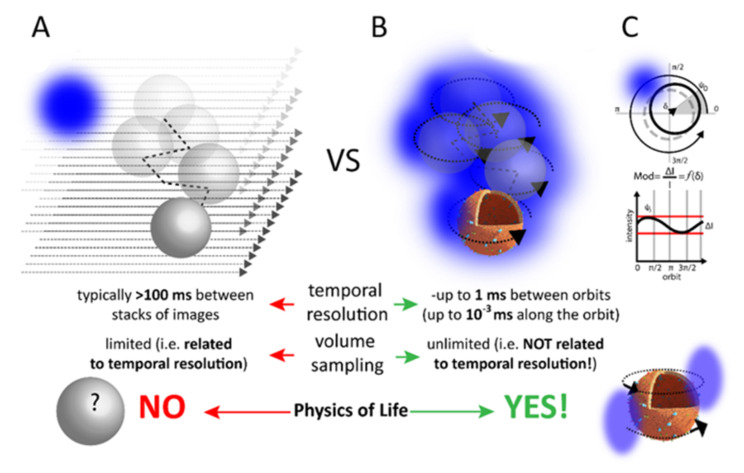
The orbital tracking approach. (**A**) The classical strategy of imaging subcellular nanostructures in 3D relies on probing the volume in a time-consuming target-search process. (**B**) The time resolution is not enough to grab (molecular) details on the structure of interest. By contrast, by forming a light envelope around the point of interest, the time resolution remains constant during the measurement (microseconds along the orbit, milliseconds in between orbits), and becomes suitable to study molecular processes on the target. (**C**) Modulation of signals detected along the orbit is kept at a minimum, i.e., the target is kept at the center of the orbit.

If any information is lost using the experimental platforms discussed so far to study granule dynamics, it is certainly related to the large amount of molecular information enclosed within these dynamic subcellular nanostructures. Such information, in fact, is inevitably averaged out during the measurement (either iMSD-based or SPT-based) due to poor temporal resolution. Theoretically, however, there is no technical limit to the possibility of retrieving molecular information, provided that sufficient acquisition speed can be achieved. To tackle this challenge, some of us are currently proposing a change of paradigm in the way these subcellular nanostructures can be imaged. In particular, we proposed to use the reference system of the single dynamic nanostructure, the single insulin granule. To this end, an excitation light-beam can be focused on a periodic orbit around the point of interest (Figure 11).

The signal recorded along the orbit is then used as feedback to localize the structure and continuously adapt the orbit position. The result is that a light envelope is formed around the structure with a temporal resolution of microseconds along the orbit and milliseconds between orbits; the typical resolutions of a scanning microscope. Of note, such temporal resolution is constant during the measurement. Once in the new reference system, fluctuation spectroscopy, by definition, will be the ideal tool to extract quantitative information with single molecule sensitivity in presence of many molecules, which is a fundamental requisite at this level. A preliminary demonstration was provided by capturing metabolism-dependent subtle fluctuations of the physicochemical properties of the solvent (i.e., polarity) in the lumen of a 3D-moving lysosome, probing their relevance both in the physiological and pathological (i.e., Krabbe disease) state of this organelle [95]. If successful, this will represent a paradigmatic shift in the way we describe biological processes within islets and β-cells, having the potential to unveil new molecular determinants of β-cell physiology and the pattern of their misregulation that is characteristic of the pathological state.
